# Regeneration Strategies in Seed Plants: A Continuum Shaping Survival

**DOI:** 10.1002/ece3.73979

**Published:** 2026-07-07

**Authors:** Keyvan Maleki, Elias Soltani

**Affiliations:** ^1^ Department of Horticulture and Crop Science The Ohio State University Columbus Ohio USA; ^2^ Department of Agronomy and Plant Breeding Sciences, College of Aburaihan University of Tehran Tehran Iran

**Keywords:** germination niche, regeneration strategies, seed banks, seed dispersal, seed dormancy

## Abstract

Seed plants dominate terrestrial ecosystems in part because regeneration is not a single event, but a coordinated sequence of traits acting across time and space. Here, we synthesize regeneration strategies in seed plants as a continuous, integrated framework spanning pre‐germination regulation, germination timing, seedling emergence, soil seed banking, seed production, and dispersal. Drawing on examples from deserts, forests, fire‐prone shrublands, wetlands, and alpine systems worldwide, we show how these stages are evolutionarily coupled into coherent regeneration syndromes that maximize fitness under distinct environmental regimes. Dormancy, serotiny, germination cue sensitivity, and bet‐hedging strategies regulate the timing of recruitment; seed size, reserve allocation, and emergence traits mediate early survival; soil seed banks and mast seeding buffer populations against temporal variability; and dispersal mechanisms determine spatial escape, colonization, and coexistence. Rather than acting independently, these traits interact to resolve fundamental trade‐offs between risk avoidance and opportunity capture, producing predictable patterns across life histories, biomes, and phylogenetic lineages. Viewing regeneration as a continuum clarifies why contrasting strategies, such as rapid germination versus prolonged dormancy, prolific seed output versus large‐seed investment, or local retention versus long‐distance dispersal, can each be adaptive depending on ecological context. We argue that this integrative perspective provides a unifying framework for understanding plant persistence, community assembly, and evolutionary diversification, and is essential for predicting seed regeneration outcomes under accelerating climate change and altered disturbance regimes.

## Graphical abstract

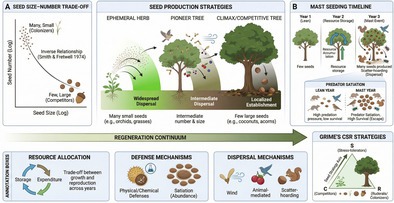

## 1 Introduction

Successful regeneration is the foundation of plant persistence, population dynamics, and evolutionary success (Walck et al. 2011; Beckman, Aslan, Rogers, Kogan, et al. 2020). In seed plants, regeneration is not a single event but a sequence of tightly linked stages—from seed production and dormancy through germination, seedling emergence, and establishment—each representing a distinct ecological filter at which failure can terminate the life cycle (Harper 1977; Laughlin 2023). However, the relative importance of each stage in determining overall regeneration success remains debated, with some studies emphasizing pre‐germination filters such as dormancy and dispersal (Donohue et al. 2010), while others argue that post‐germination processes including seedling establishment represent the primary demographic bottleneck (Grubb 1977; Beckman, Aslan, and Rogers 2020). This lack of consensus reflects both genuine biological context‐dependence and persistent gaps in comparative, multi‐stage demographic data.

Historically, regeneration traits have often been studied in isolation (Saatkamp et al. 2019). Seed dormancy has been examined as an adaptation to environmental unpredictability, germination cues as mechanisms ensuring seasonal or microsite matching, seed banks as demographic buffers against disturbance, seed production as an allocation trade‐off between offspring quantity and quality, and dispersal as a means of escaping competition, enemies, or unsuitable habitats (Willis et al. 2014; Beckman, Aslan, Rogers, Kogan, et al. 2020; Rosbakh et al. 2023). While this body of work has yielded deep insights, it has also fostered a fragmented view of regeneration, in which individual traits are emphasized without fully considering their functional integration across life‐cycle stages (Pausas et al. 2024). Although it is increasingly argued that regeneration success emerges from coordinated trait syndromes rather than single traits acting alone (Harper 1977; Saatkamp et al. 2019), empirical support remains uneven—some studies identify coherent regeneration syndromes linking seed size, dormancy class, and seed bank persistence (Saatkamp et al. 2019), while others find regeneration traits to be weakly correlated or highly context‐dependent (Díaz et al. 2016; Laughlin 2023). Whether trait coordination reflects shared selective pressures, developmental constraints, or phylogenetic conservatism remains a major unresolved challenge in regeneration ecology.

Despite growing recognition of these patterns, no existing synthesis explicitly integrates all major regeneration stages—pre‐germination strategies, germination behavior, seedling emergence, seed banking, seed production, and dispersal—into a single unified framework applicable across biomes and life forms. Most reviews address one or two stages, often within particular ecosystems or taxonomic groups, limiting cross‐system comparison (Leck et al. 2008; Violle et al. 2014; Liu and Ma 2015; Saatkamp et al. 2019). Furthermore, two axes of pre‐germination strategy—seed water absorption dynamics and post‐dispersal embryo growth—have received little synthetic attention despite their ecological significance. Such integration is increasingly urgent given that climate warming, altered disturbance regimes, habitat fragmentation, and shifting species interactions are all expected to act strongly and differentially across regeneration stages (Walck et al. 2011), yet predicting regeneration responses to global change remains limited by the absence of multi‐stage frameworks that capture how constraints at one stage cascade forward to shape outcomes at others.

Here, we present a unified framework for regeneration strategies in seed plants, framing regeneration as a continuous, multi‐stage process in which trade‐offs at one stage constrain or enable strategies at subsequent stages and convergent solutions recur across lineages and biomes. Drawing on examples from deserts, grasslands, tropical and temperate forests, fire‐prone ecosystems, and alpine environments, we integrate ecological, evolutionary, and physiological perspectives across the entire regeneration cycle. We review (1) pre‐germination strategies, including dormancy, serotiny, and an overlooked axis of seed water absorption and post‐dispersal embryo growth (2) germination strategies and environmental cueing; (3) seedling emergence and the role of soil seed banks; (4) seed production strategies and reproductive allocation; and (5) dispersal mechanisms and spatial dynamics. We acknowledge that this framework is necessarily a simplification—vegetative reproduction, clonal spread, and apomixis fall outside its scope—and that our synthesis draws disproportionately on temperate and Mediterranean systems, with gaps in other biomes flagged throughout. By synthesizing regeneration traits within a unified continuum, this review aims to advance a more integrative understanding of plant regeneration ecology and to inform predictions of species responses to ongoing global change and strategies for conservation and restoration.

## 2 Pre‐Germination Strategies

### 2.1 Seed Dormancy

**FIGURE 1:**
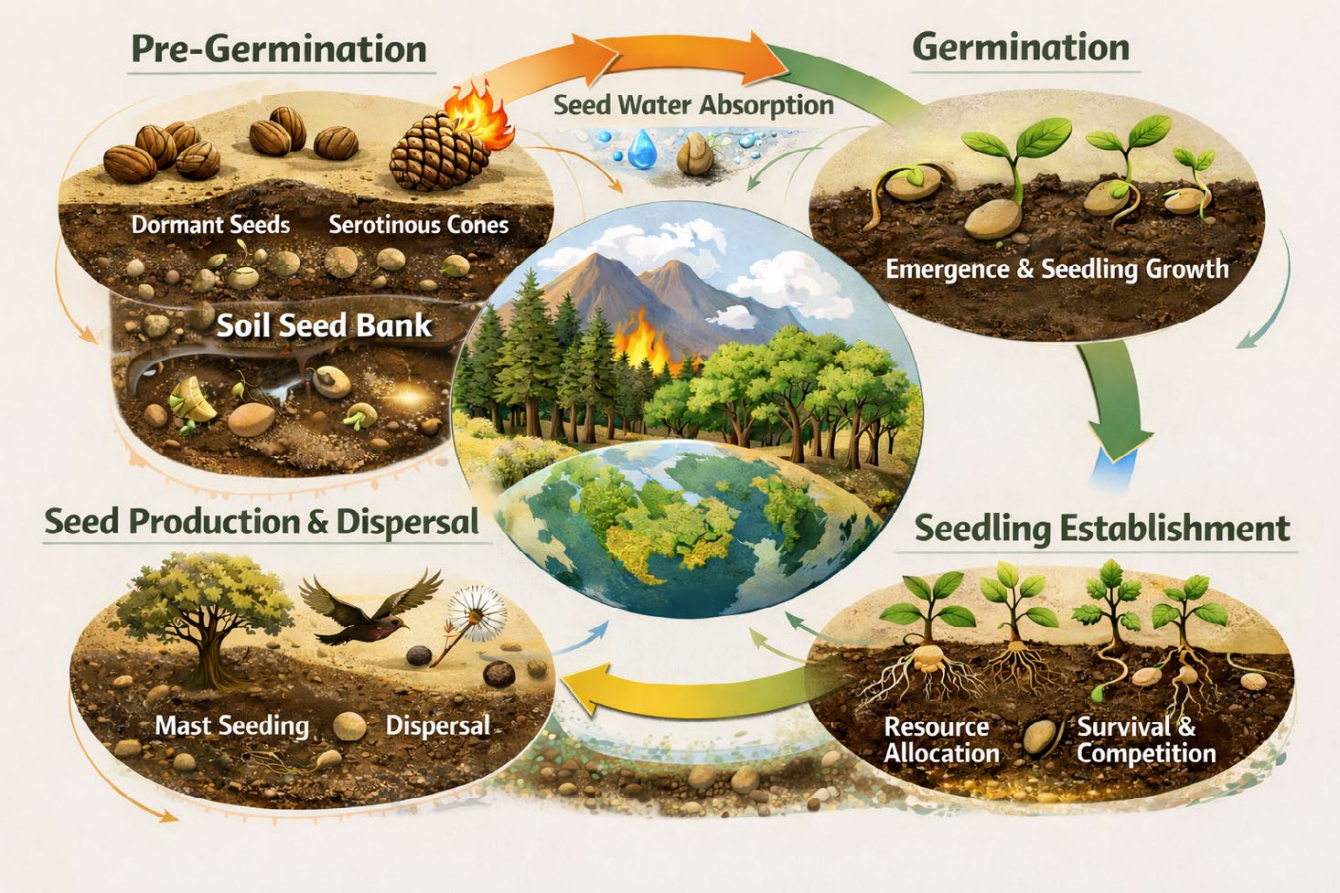
Conceptual framework of the seed regeneration continuum. Schematic illustration of regeneration in seed plants as a coordinated sequence of processes linking timing, establishment, and spatial dynamics. Pre‐germination traits, including dormancy, serotiny, soil seed banks, and seed water absorption, regulate when seeds become developmentally active in response to environmental cues. Germination initiates emergence and early seedling growth, while subsequent seedling establishment is shaped by reserve allocation, survival, and competitive interactions. Seed production strategies (e.g., seed size–number trade‐offs and mast seeding) determine propagule supply, and dispersal mediates spatial escape, colonization, and population persistence across heterogeneous landscapes.

Prior to germination, many seed plants employ dormancy and other pre‐germination strategies to delay or control the onset of establishment (Baskin and Baskin 2014). Within the regeneration continuum, the pre‐germination stage represents the first major ecological filter determining when seeds become developmentally responsive to environmental cues (Table 1; Figure 1). Seed dormancy is widely regarded as an adaptive trait that prevents seeds from germinating under unfavorable or suboptimal conditions, though the extent to which dormancy is directly adaptive versus a developmental constraint or phylogenetic legacy remains debated (Finch‐Savage and Leubner‐Metzger 2006; Jurado and Flores 2005). By temporarily halting germination, dormancy ensures that seedling emergence is synchronized with seasons or microhabitats that enhance survival (Maleki et al. 2022). For example, in temperate regions, seeds with physiological dormancy often require a prolonged cold period (winter) before they can germinate, thereby ensuring emergence in spring rather than autumn (Baskin and Baskin 2014). In deserts or unpredictable climates, dormancy is crucial for avoiding germination after small rain events that would not sustain seedlings (Gremer and Venable 2014). Indeed, dormancy is considered a bet‐hedging strategy: seeds remain quiescent until the probability of seedling success is high, spreading the risk of failure across time (Gremer and Venable 2014; Pausas et al. 2022; Abley et al. 2024). Dormancy prevalence tends to increase in more unpredictable environments—Baskin and Baskin (2014) estimated that as many as 85% of arid‐zone species produce dormant seeds—though this estimate should be interpreted cautiously given potential taxonomic sampling biases and variation across dormancy classification systems (Rosbakh et al. 2023).

**TABLE 1:** Major pre‐germination strategies in seed plants and their ecological functions.

Strategy	Mechanistic basis	Primary environmental context	Taxonomic distribution	Ecological function (what it regulates)	Adaptive significance
Physiological dormancy (PD)	Hormonal inhibition (ABA–GA balance); dormancy release requires environmental cues (e.g., cold stratification, after‐ripening)	Seasonal climates (temperate), also common in unpredictable environments	Widespread; present across major angiosperm clades and many gymnosperms	Timing of germination via delaying metabolic commitment until cues indicate favorable season	Synchronizes emergence with favorable windows; reduces “wrong‐season” emergence; supports bet‐hedging
Physical dormancy (PY)	Water‐impermeable seed coat; broken by scarification (abrasion, fire heat, gut passage, microbes)	Fire‐prone, seasonal, arid and semi‐arid systems	Common in Fabaceae and multiple angiosperm families	Controls imbibition (blocks water uptake) until disturbance cue occurs	Prevents germination after inadequate moisture pulses; links recruitment to disturbance‐created opportunity
Morphological dormancy (MD)	Underdeveloped embryo at dispersal; embryo grows under favorable hygrothermal conditions until reaching threshold size	Broad; often seasonal systems where growth can proceed when conditions permit	Widespread across angiosperms (notably in several temperate lineages)	Controls germination timing by requiring intraseminal embryo growth before radicle emergence	Prevents immediate germination; aligns completion of development with favorable conditions
Morphophysiological dormancy (MPD)	Underdeveloped embryo + physiological block; requires stratification regimes (warm, cold, or sequences) before embryo growth proceeds	Strongly seasonal climates; species‐specific regimes tuned to local seasonality	Common in temperate floras; multiple dormancy “levels” (e.g., deep/simple/complex)	Sequential environmental filtering: first physiological release, then embryo growth	High precision timing; discriminates transient favorable spells from true seasonal transitions
Post‐dispersal embryo growth as a timing strategy (MD/MPD continuum)	Embryo elongation proceeds only when hydric and thermal requirements are met; in MPD, ABA/GA balance and stratification can gate embryo growth	Seasonal environments; climates where timing errors are costly	Many taxa with underdeveloped embryos (often E:S < 0.5 at dispersal)	Controls phenology of germination through developmental “checkpoint” before radicle emergence	Aligns recruitment with optimal establishment seasons; allows multi‐signal integration while in soil/seed bank
Seed water uptake (imbibition) strategies *(new axis)*	Variation in imbibition rate/capacity and sensitivity to water potential; driven by coat permeability, surface‐area: volume, mucilage, cell wall/testa properties	Arid/semi‐arid (avoid “false starts”); pulse‐driven systems; also relevant in most habitats as the first filter	Likely widespread but poorly synthesized across clades/biomes	Hydration gating before dormancy release and germination progression; can function analogously to dormancy	Avoids commitment after transient moisture; enables rapid exploitation of short moisture windows; shapes regeneration niche differentiation
Mucilage‐mediated hydration buffering *(imbibition‐linked)*	Hydrophilic polysaccharides swell upon wetting, altering boundary‐layer moisture and retention; can mediate microbe interactions	Drying‐prone soils; surface germination; saline or crusted substrates (often)	Multiple angiosperm groups	Microenvironmental engineering: retains water, buffers desiccation risk, may influence microbes	Stabilizes hydration during early metabolic reactivation; can increase establishment probability under variable moisture
Serotiny (canopy seed storage)	Seeds retained on plant in closed cones/fruits; release triggered by fire heat or drought	Fire‐prone Mediterranean, savanna, boreal systems	Several gymnosperm groups (e.g., Pinaceae) + ≥ 12 angiosperm families	Delays seed release until post‐disturbance window	Mass release into reduced competition + nutrient flush; enhances recruitment after fire
Long‐term soil seed banking	Seeds persist viable in soil (transient vs. persistent banks) via dormancy + resistance traits	Disturbance‐driven, arid, seasonal and stochastic systems	Widespread across angiosperms	Spreads recruitment through time; maintains “reserve regeneration”	Buffers multi‐year recruitment failure; supports population persistence through unfavorable periods
Recalcitrant seeds (nondormant, desiccation‐sensitive)	Shed in germination‐ready state; low desiccation tolerance; poor longevity	Humid tropical forests; reliably moist environments	~8% globally; higher in humid tropics (~18.5%)	Minimizes delay—rapid transition to seedling stage	Favors immediate establishment where moisture is predictable

Dormancy can be imposed by hard seed coats (physical dormancy), as found in many legumes and other angiosperms, which prevent water uptake until the coat is scarified by abrasion, fire, or passage through an animal's gut (Table 1). In several plant families, an underdeveloped embryo is another reason for seed dormancy, and these seeds need a period of time for post‐dispersal embryo growth. It can also be maintained by physiological mechanisms (hormonal balances that inhibit germination until certain cues occur); notably, physiological dormancy is the most common dormancy class and is present in all major clades of angiosperms as well as in many gymnosperms (Baskin and Baskin 2014). Overall, the prevalence of dormancy across seed plants is consistent with adaptive significance, though causality is difficult to establish conclusively. Several alternative explanations exist: dormancy may in some cases reflect developmental constraints rather than direct selection, or may be maintained by genetic linkage with other selected traits (Donohue et al. 2010). Furthermore, while dormancy clearly reduces germination in unfavorable conditions, the degree to which it actually improves long‐term fitness has been demonstrated empirically in only a limited number of species and systems (Gremer and Venable 2014).

### 2.2 Seed Water Uptake as an Overlooked Axis of Regeneration Strategy

Beyond dormancy *sensu stricto*, seed water absorption (imbibition) represents a fundamental but comparatively underappreciated pre‐germination trait that strongly influences regeneration outcomes (Table 1). Imbibition is the initial physical process by which dry seeds absorb water upon exposure to moisture, triggering metabolic reactivation and setting the pace for subsequent developmental transitions (Nonogaki et al. 2010; Bewley et al. 2013). Although imbibition is a prerequisite for radicle emergence, it does not necessarily lead to dormancy release or germination—in physiologically and morphophysiologically dormant seeds, full water uptake may occur while hormonal or developmental blocks maintain quiescence (Bewley et al. 2013; Baskin and Baskin 2014). Imbibition dynamics nevertheless act as a critical physiological filter, determining whether seeds are even capable of responding to subsequent dormancy‐breaking cues, and should therefore be understood as a necessary but not sufficient condition for germination rather than a direct trigger for dormancy release. Variation in imbibition rate and capacity reflects both seed structural traits (e.g., seed coat thickness, permeability, surface area‐to‐volume ratio) and biochemical properties (e.g., cell wall composition, mucilage production), which together regulate water uptake under different environmental conditions (Smýkal et al. 2014; Steinbrecher and Leubner‐Metzger 2018). Seeds with highly permeable coats may imbibe rapidly, allowing fast metabolic activation and germination when moisture availability is brief, whereas seeds with restrictive coats may exhibit slow or threshold‐dependent water uptake, effectively delaying germination until sustained hydration occurs. In physically dormant seeds, imbibition and dormancy release are tightly coupled—water entry is prevented until structural barriers are breached—but in physiologically or morphophysiologically dormant seeds, full imbibition may occur while germination remains blocked by hormonal or developmental constraints (Finch‐Savage and Leubner‐Metzger 2006; Bewley et al. 2013). Recognizing this distinction is essential for understanding how imbibition dynamics interact with different dormancy mechanisms across the regeneration continuum.

**FIGURE 2:**
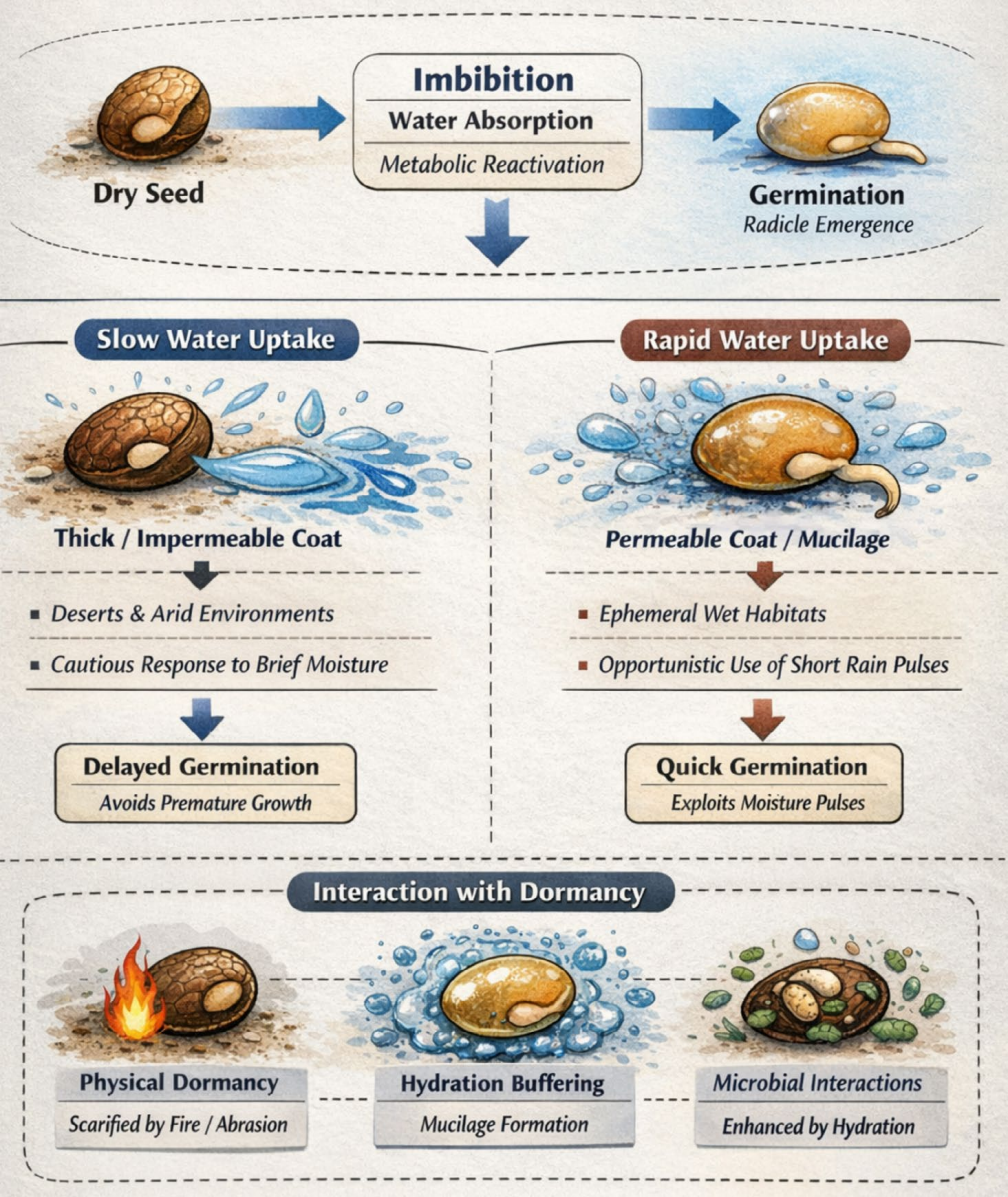
Seed water uptake (imbibition) as a hidden axis of regeneration strategy. Imbibition represents the initial physiological transition from dry seed to germination, mediating metabolic reactivation prior to radicle emergence. Variation in water uptake dynamics, ranging from slow, restricted imbibition associated with thick or impermeable seed coats to rapid imbibition facilitated by permeable coats or mucilage production, shapes regeneration outcomes across environments. In arid and unpredictable systems, slow or threshold‐dependent water uptake can delay germination and reduce the risk of premature establishment following brief moisture pulses. Conversely, rapid imbibition enables opportunistic germination in habitats characterized by short but predictable rainfall events. Imbibition dynamics also interact with other pre‐germination mechanisms, including physical dormancy (broken by fire or abrasion), mucilage‐mediated hydration buffering, and microbially mediated processes. Together, these pathways highlight seed water absorption as an ecologically meaningful and environmentally structured trait that operates alongside dormancy to regulate regeneration timing. This figure was created using the free version of BioRender.

Emerging evidence suggests that imbibition traits may be adaptive and environmentally structured, though this hypothesis remains largely untested at broad comparative scales. Much of the existing evidence comes from agricultural species or controlled laboratory experiments, which may not accurately reflect the diversity of imbibition strategies in natural plant communities (Bewley et al. 2013). Furthermore, because imbibition rate is influenced by multiple structural and biochemical seed traits simultaneously, isolating its adaptive significance independent of other co‐varying traits remains methodologically challenging. In arid and semi‐arid environments, slower or more controlled water uptake may prevent seeds from committing to germination after small, transient rainfall events that are insufficient to support seedling establishment, though this hypothesis is currently based on preliminary unpublished observations (Maleki and Soltani, unpublished data) and awaits confirmation through controlled experimental studies across a broader range of species and environmental conditions. In habitats where moisture pulses are short but predictable, rapid imbibition may confer an advantage by enabling immediate exploitation of favorable conditions (Kos and Poschlod 2007; Gremer and Venable 2014). These contrasts suggest that imbibition rate and sensitivity to water potential may represent important axes of regeneration niche differentiation, though whether observed interspecific variation reflects local adaptation, phenotypic plasticity, or incidental variation in seed morphology remains unclear (Steinbrecher and Leubner‐Metzger 2018). Seed water absorption strategies are also closely linked to other pre‐germination traits. Physical dormancy imposed by hard seed coats directly constrains imbibition until structural barriers are breached by fire, abrasion, microbial activity, or gut passage (Smýkal et al. 2014; Baskin and Baskin 2014; Figure 2). Similarly, mucilaginous seeds, which produce hydrophilic polysaccharides upon wetting, can modify the immediate hydration environment around the seed, enhancing water retention, buffering against desiccation, and potentially facilitating microbial interactions (Western 2012; Figure 2). Such traits blur the boundary between seed structure and microenvironmental engineering, reinforcing the idea that imbibition is an ecologically meaningful component of regeneration strategy.

Despite its central role, seed water absorption remains poorly synthesized at macroecological and macroevolutionary scales (Table 1). Most existing studies focus on individual species, functional groups, or agricultural taxa, often under controlled laboratory conditions, with little comparative integration across biomes or phylogenetic lineages (Bewley et al. 2013; Steinbrecher and Leubner‐Metzger 2018; Miano et al. 2018). To date, no global analysis has systematically quantified variation in seed imbibition dynamics across growth forms, climates, or evolutionary histories, nor evaluated how imbibition traits covary with dormancy class, seed size, or germination niche breadth. This gap is particularly consequential in the context of global change. Altered precipitation regimes, increased rainfall variability, and shifts in soil moisture dynamics are expected to act directly on early hydration processes, potentially generating mismatches between evolved imbibition strategies and contemporary moisture regimes (Walck et al. 2011; Saatkamp et al. 2019). Integrating seed water absorption into regeneration frameworks therefore represents a critical frontier for understanding plant persistence under changing climates.

### 2.3 Post‐Dispersal Embryo Growth as a Timing Strategy for Seed Regeneration

**FIGURE 3:**
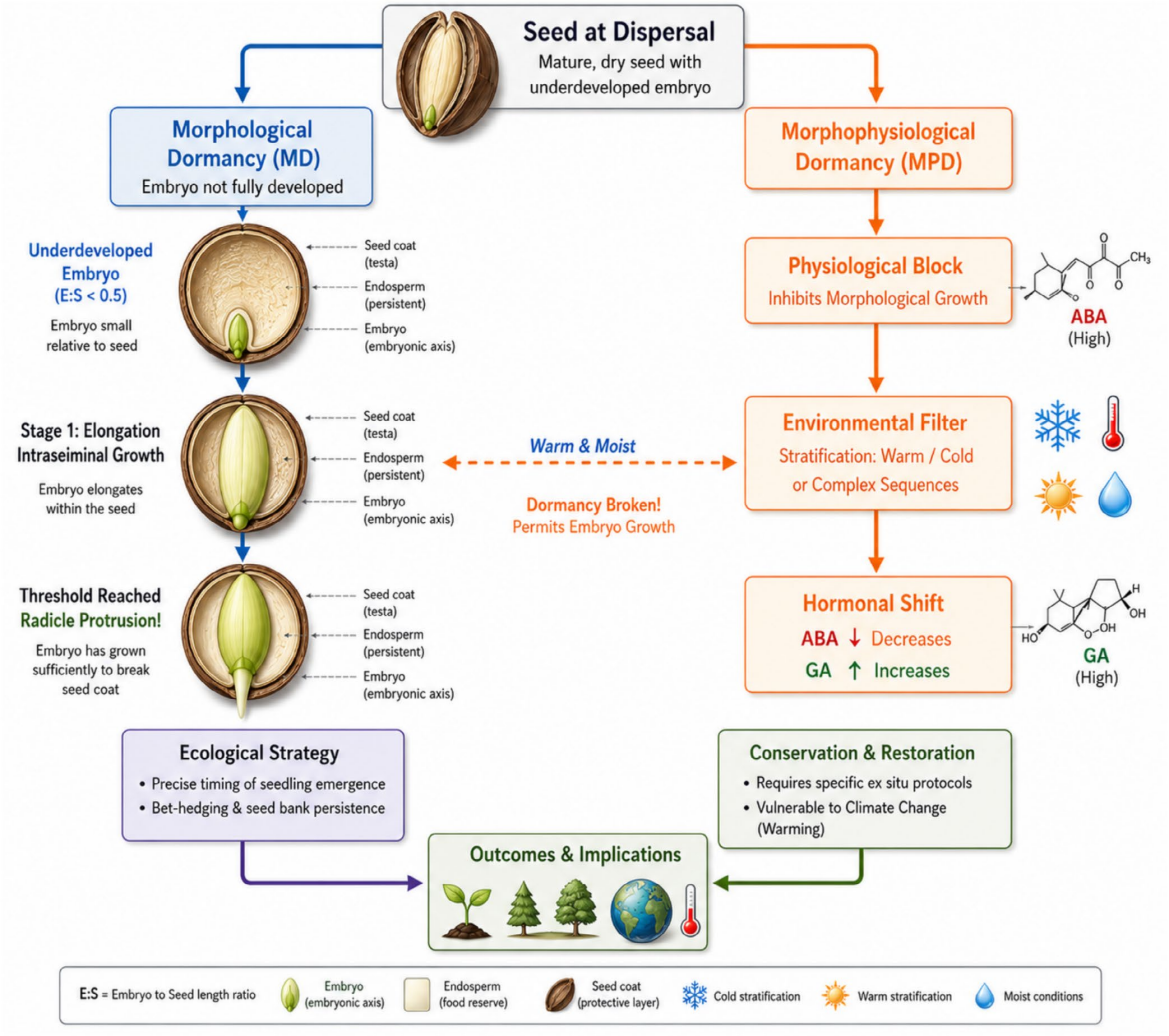
Post‐dispersal embryo growth in seeds with morphological (MD) and morphophysiological dormancy (MPD). Schematic comparing the MD (blue) and MPD (orange) pathways from dispersal to germination. A green embryo is shown with its internal elongation. At dispersal, both MD and MPD seeds contain an underdeveloped embryo (E:S < 0.5). Under favorable conditions, MD seeds undergo simple intraseminal growth until the embryo reaches the threshold size for germination. In MPD seeds, an additional physiological block requires complex stratification sequences (e.g., warm → cold) to be alleviated before embryo growth can proceed. The embryo‐to‐seed ratio (E:S) progresses from ~0.4 to 1.0 during elongation and germination. Environmental signals regulate this process through molecular controls (ABA/GA balance), with important implications for conservation, restoration, and climate change vulnerability. ABA, abscisic acid; E:S, Embryo‐to‐Seed ratio; GA, gibberellins. This figure was created using the free version of BioRender.

Seeds of a wide range of species are dispersed with differentiated but underdeveloped embryos, requiring post‐dispersal embryo growth before germination can occur (Forbis et al. 2002; Baskin and Baskin 2014; Table 1). This condition is formally categorized as morphological (MD) or morphophysiological dormancy (MPD) (Baskin and Baskin 2004; Finch‐Savage and Leubner‐Metzger 2006). Within the regeneration continuum, embryo growth functions as a pre‐germination timing mechanism that links dormancy status to subsequent germination and seedling emergence (Donohue et al. 2010; Walck et al. 2011). In MD seeds, the embryo initiates expansion upon exposure to favorable hygrothermal conditions (Figure 3). Germination is only finalized once the embryo reaches a species‐specific threshold length, leading to radicle protrusion (Hidayati et al. 2000; Vandelook et al. 2007). In MPD seeds, embryo growth is additionally blocked by a physiological component that must be alleviated, often by seasonal temperature cues, before morphological growth can proceed (Baskin and Baskin 2004; Copete et al. 2011; Bewley et al. 2013). Although the diversity of MPD types is often interpreted as an adaptation for precise seasonal timing, direct evidence that they are finely tuned by natural selection to local environments remains limited because most inferences come from ex situ stratification experiments whose relevance to natural soil conditions is still uncertain (Baskin and Baskin 2004; Finch‐Savage and Footitt 2017).

The physiological release of dormancy in MPD seeds is governed by diverse stratification regimes tailored to seasonal cycles (Baskin and Baskin 2004, 2014; Geneve 2003; Figure 3). Depending on the species and its ecological origin, dormancy break may require cold stratification, warm stratification, or sequential combinations of both, including warm‐cold, cold‐warm, or repeated temperature cycles (Finch‐Savage and Leubner‐Metzger 2006; Bewley et al. 2013; Zardari et al. 2019; Herranz et al. 2010; Phartyal et al. 2009; Baskin and Baskin 2003; Copete et al. 2011; Walck et al. 1999; Kondo et al. 2002; Vandelook et al. 2007). In deep complex MPD, for example, sequences such as warm‐cold‐warm can prevent premature germination during transient winter warming and align embryo elongation with the onset of the most favorable growing season (Donohue et al. 2010; Fenner and Thompson 2005; Walker et al. 2025; Baskin and Baskin 2004, 2014; Phartyal et al. 2009; Copete et al. 2011; Zardari et al. 2019; Herranz et al. 2010). These patterns support the view that post‐dispersal embryo growth acts as an environmental filter and sensing mechanism rather than a simple developmental delay (Finch‐Savage and Footitt 2017; Blandino et al. 2025), yet linking specific stratification sequences to fitness outcomes in natural populations remains difficult, and intraspecific variation in thermal requirements is rarely incorporated into broad species‐level generalizations (Rosbakh et al. 2023).

The duration of post‐dispersal embryo growth varies considerably among species, populations, initial embryo length, and dormancy types (Hidayati et al. 2000; Vandelook et al. 2007; Soltani et al. 2019; Hashemirad et al. 2023; Blandino et al. 2025). In simple MD, embryo growth may be completed within days to weeks under continuously warm and moist conditions, whereas deep simple MPD may require weeks to months of stratification before morpho‐anatomical development can proceed (Forbis et al. 2002; Baskin and Baskin 2004; Baskin and Baskin 2014). This temporal variation creates distinct germination phenologies, from autumn germination following summer dispersal to delayed emergence in the subsequent spring (Donohue 2005; Cochrane et al. 2015). By extending the pre‐germination phase, embryo growth can also interact with soil seed bank dynamics, allowing seeds to remain viable while integrating multiple environmental signals, including temperature, moisture, and photoperiod (Fenner and Thompson 2005; Donohue et al. 2010; Thompson et al. 1997; Ooi 2012; Long et al. 2015; Baskin and Baskin 2014; Finch‐Savage and Footitt 2017). Its proposed role as a bet‐hedging mechanism, however, remains largely theoretical, because empirical evidence that variation in embryo growth rates increases fitness through temporally spread germination is still scarce under field conditions (Simons 2011).

Recent molecular and physiological studies have begun to elucidate the regulatory mechanisms controlling embryo growth in dormant seeds (Bewley et al. 2013; Nonogaki 2014). Although most molecular studies on hormonal regulation of dormancy have focused on species with physiological dormancy (PD), the balance between abscisic acid (ABA) and gibberellins (GAs) is widely considered to play a pivotal role in modulating dormancy and germination across dormancy classes (Finkelstein et al. 2008; Graeber et al. 2012). In MPD seeds, exogenous GA application can often substitute for stratification requirements and promote embryo elongation, suggesting that a similar ABA/GA regulatory framework operates in these species (Mattana et al. 2012; Baskin and Baskin 2014; Walker et al. 2025). However, direct molecular evidence for ABA/GA dynamics in MPD seeds remains limited, and findings from model species with physiological dormancy may not translate directly to MPD species, which involve different developmental constraints (Graeber et al. 2012). This uncertainty has practical implications for conservation and restoration because many threatened species exhibit MD or MPD and failure to match embryo growth requirements can lead to poor germination and establishment in ex situ and restoration programs (Walck et al. 2011; Baskin and Baskin 2014; Maleki, Chmielarz, et al. 2024; Maleki, Soltani, et al. 2024; Zhang et al. 2023; Broadhurst et al. 2016). Climate change may further desynchronize temperature cues, embryo development, and establishment windows, making this stage critical for understanding how pre‐germination timing shapes later regeneration success and population persistence (Walck et al. 2011; Mondoni et al. 2011; Fernández‐Pascual et al. 2019).

### 2.4 Serotiny

In addition to dormancy per se, some plants have evolved prolonged seed storage on the mother plant (so‐called serotiny) as a pre‐germination strategy (Keeley and Pausas 2022). Within the regeneration continuum, serotiny functions as an external timing mechanism in which seed release, rather than seed dormancy status or germination physiology, is controlled by environmental triggers. Serotiny refers to the retention of mature seeds in closed cones or fruits on the plant until an environmental trigger induces their release (Lamont et al. 2020). While fire has long been considered the primary selective driver of serotiny, this view has been increasingly challenged. Keeley and Pausas (2022) argued that drought stress may be an equally or more important driver in some lineages, as cone opening in response to desiccation can enhance dispersal during dry periods regardless of fire. This debate has important implications for predicting how serotinous species will respond to changing fire regimes and altered precipitation patterns under climate change, as fire‐adapted and drought‐adapted serotiny may have fundamentally different ecological consequences. This strategy is best known in fire‐prone ecosystems: for instance, many *Pinus* species (gymnosperms) in Mediterranean‐climate shrublands, savannas, or boreal forests have serotinous cones sealed with resin that melt and open only during the intense heat of a wildfire (Lamont et al. 1991; Vincenzi and Piotti 2014; Keeley and Pausas 2022). The adaptive benefit of post‐fire seed release is intuitive—seeds fall onto a landscape with reduced competition and nutrient‐rich ash—but empirical quantification of the fitness advantage of serotiny over nonserotiny in fire‐prone systems has proven difficult. The benefit depends critically on fire return intervals, and when fires occur too frequently or too infrequently relative to plant maturation time, serotiny may actually reduce rather than enhance fitness (Lamont et al. 2020). Furthermore, serotiny's advantage assumes that post‐fire conditions reliably favor seedling establishment, which is increasingly uncertain under climate‐driven changes in post‐fire moisture availability (Pausas et al. 2022). The seeds can then germinate in an environment with reduced competition and ample resources. Serotiny is widespread in fire‐prone regions (documented in at least 12 families of angiosperms and several gymnosperm groups) and shows a continuum from “weak” serotiny (cones open after a few years even without fire) to “strong” serotiny (cones remain sealed for a decade or more) (Lamont et al. 2020). It has been observed in Mediterranean‐type ecosystems of Australia, South Africa, North America, and the Mediterranean Basin, among others (Lamont et al. 2020). The strategy provides a fitness advantage in fire regimes where fire‐return intervals are shorter than the plant's lifespan, seeds are stored until fire creates favorable conditions, then a massive, synchronized release occurs. This greatly increases the availability of seeds for population recovery after fire, beyond what would be available if seeds were released and germinated in inter‐fire periods. In effect, serotiny is another form of dormancy, but one external to the seed's physiology: the environment (fire) cues the parent plant to release seeds only at the right moment. Other pre‐germination strategies include long‐term seed viability in the soil or on plant (some seeds can remain viable for decades waiting for a trigger), and morphological features like tough seed coats or waxy endocarps that enforce dormancy.

### 2.5 Recalcitrant Seeds and Immediate Germination Strategies

At the opposite end of the pre‐germination continuum from dormancy and long‐term seed storage, some species minimize the delay between dispersal and germination. Recalcitrant seeds, common in many tropical angiosperms (e.g., coconut, cacao, many mangroves) and some temperate trees (oak, chestnut), are desiccation‐sensitive and cannot withstand substantial drying or prolonged storage; as a result, they typically germinate quickly after dispersal or lose viability (Daws et al. 2005; Baskin and Baskin 2014). In contrast, orthodox seeds tolerate drying to low moisture contents, can often remain viable for long periods in storage, and are much more likely to form persistent soil seed banks that buffer regeneration through time (Baskin and Baskin 2014; Probert et al. 2009). This difference in storage physiology has major ecological consequences. Orthodox seeds are generally favored in seasonal, disturbance‐prone, or environmentally unpredictable habitats, where delayed germination and temporal buffering enhance fitness, whereas recalcitrant seeds are more common in reliably moist environments where the costs of delayed germination may exceed the benefits of dormancy or long‐term persistence (Daws et al. 2005).

Globally, recalcitrant seeds are estimated to occur in about 8% of seed plant species, but in humid tropical forests this proportion rises to ~18.5% of species (Daws et al. 2005). However, these estimates should be treated with caution, as the distinction between recalcitrant and orthodox seeds is itself a simplification of a broader continuum of seed storage behavior, including intermediate seeds, and the global dataset underpinning these estimates is heavily biased toward economically important species with agricultural or horticultural relevance (Probert et al. 2009). Because recalcitrant seeds are short‐lived and generally unable to persist in the soil, they rarely contribute to long‐term seed‐bank formation and instead depend on immediate establishment or continuous seed rain for population persistence (Baskin and Baskin 2014). Immediate germination may therefore represent an alternative solution to the timing problem addressed by dormancy in other systems, although whether this pattern reflects direct selection for rapid establishment or passive tolerance of a physiological limitation remains unresolved. Framed this way, recalcitrant and orthodox seeds represent contrasting solutions to regeneration in time: one emphasizing rapid establishment under favorable conditions, the other emphasizing persistence, delay, and demographic buffering. Whether via enforced dormancy or immediate germination, the pre‐germination phase is thus a critical filter that aligns the plant life cycle with environmental opportunity.

## 3 Germination Strategies

**FIGURE 4:**
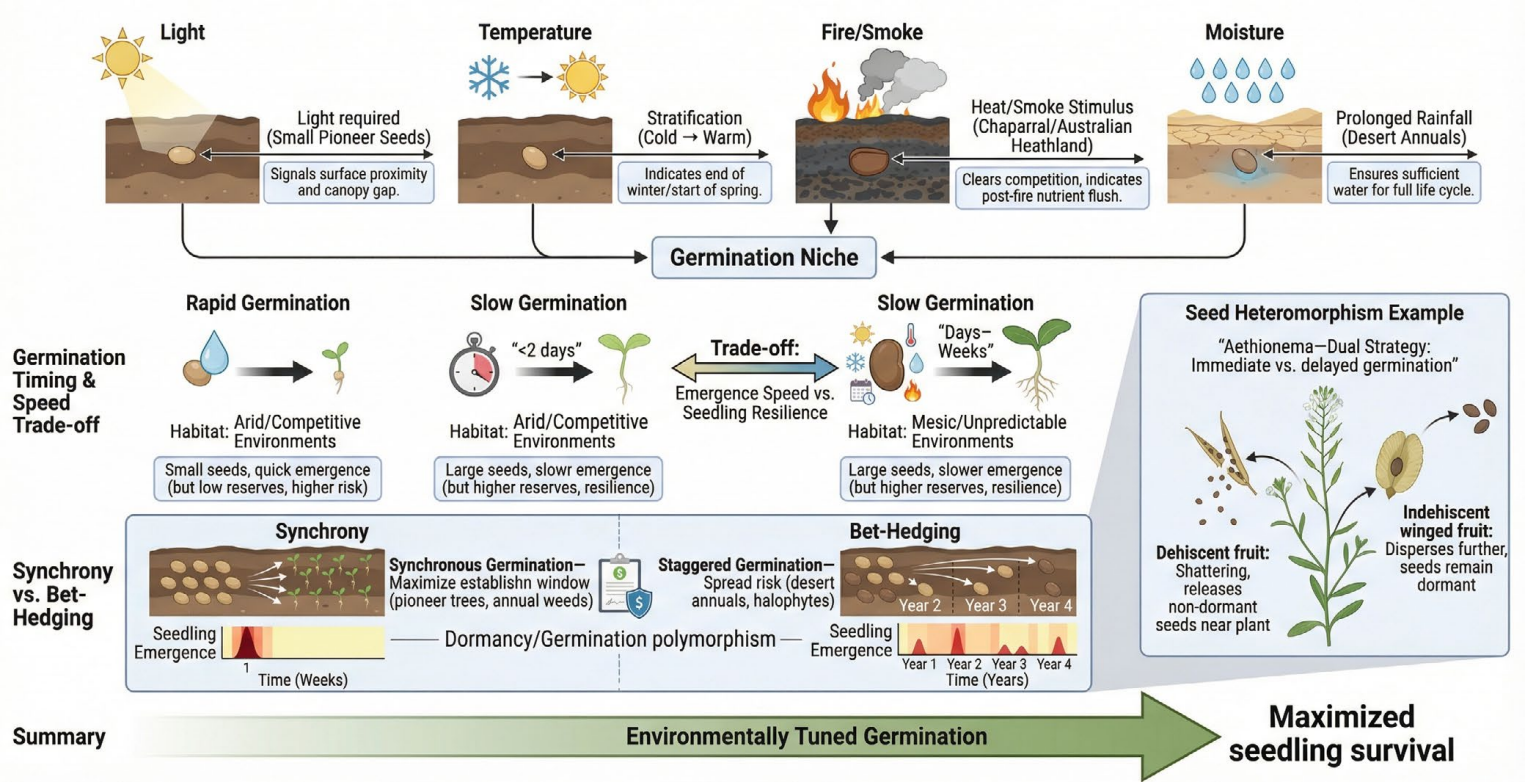
Conceptual framework for the germination niche: Environmental cueing, germination phenology, and risk‐spreading strategies. The figure summarizes how key environmental signals, light, temperature (stratification), fire/smoke, and moisture define species‐specific germination niches by regulating when seeds transition from quiescence to germination. These cues interact with variation in germination timing and speed, illustrating a central trade‐off between rapid germination that exploits brief establishment windows and slower germination associated with greater seedling resilience (often linked to larger seed reserves). The lower panel contrasts synchronous germination, which can maximize establishment during short favorable periods, with staggered (fractional) germination as a bet‐hedging strategy that spreads recruitment risk across years in unpredictable environments. A schematic example of seed heteromorphism (e.g., Aethionema) highlights how a single species can partition regeneration in space and time via distinct dispersal and dormancy phenotypes. Collectively, these pathways show how environmentally tuned germination strategies can enhance recruitment success and maximize seedling survival across variable habitats. This figure was created using the free version of BioRender and Figurelabs.

Germination represents a pivotal transition in the regeneration continuum—the point at which a seed commits irreversibly to development and becomes vulnerable to environmental conditions as a seedling (Figure 4). It is important to distinguish germination strategy from dormancy regulation: dormancy determines whether and when a seed becomes capable of germinating in response to environmental cues, while germination strategy concerns how seeds exploit those cues once dormancy is released, including the speed, synchrony, and environmental thresholds that govern the germination event itself (Ten Brink et al. 2020; Gremer et al. 2020). Many environmental signals—including temperature, light, smoke, heat shocks, and rainfall thresholds—can function both as dormancy‐breaking cues and as direct germination triggers, and their roles in dormancy release are discussed in the pre‐germination section. Here, we focus on how germination traits, once the seed is capable of germinating, contribute to regeneration success across environments (Fernández‐Pascual et al. 2021; Carta et al. 2022; Pausas et al. 2022).

Seeds exhibit physiological responses to environmental signals that collectively define a germination niche for each species (Fernández‐Pascual et al. 2021; Carta et al. 2022; Maleki, Chmielarz, et al. 2024; Maleki, Soltani, et al. 2024; Figure 4). For example, small‐seeded pioneer species often require light for germination (Maleki et al. 2025), ensuring that germination is restricted to open conditions near the soil surface where seedling survival is more likely. In fire‐prone and arid ecosystems, heat, smoke, and rainfall thresholds function as environmental filters that align germination with post‐disturbance or post‐rainfall windows of opportunity, as discussed in the pre‐germination section (Keeley and Pausas 2022; Gutterman 2012). By coupling germination to such reliable environmental signals, plants reduce the risk of emerging into conditions where seedlings would likely die (Pausas et al. 2022). Strong selective pressure therefore exists for mechanisms that fine‐tune germination timing, because germination under suboptimal conditions can result in enormous seedling mortality (Ten Brink et al. 2020).

Beyond the question of when to germinate, seed plants also differ in how quickly and synchronously they germinate once conditions are suitable. Rapid germination is often advantageous in habitats where favorable conditions are brief or highly competitive (Parsons 2012; Kadereit et al. 2017). Many plants in arid Australia, for example, have seeds that can go from dry to germinated within 1–2 days of rainfall, allowing seedlings to exploit soil moisture before it evaporates (Salazar et al. 2011; Duncan et al. 2019). A comparative study in the Australian arid zone showed that most species tested had nondormant, fast‐germinating seeds, with only a minority retaining dormancy, indicating that rapid germination allows arid species to capitalize on sporadic rainfall while dormancy, when present, serves to delay germination until a truly substantial rain event occurs (Duncan et al. 2019; Escobar et al. 2021). In more mesic or predictable environments, extremely rapid germination is less critical and other factors such as frost avoidance dominate germination strategy.

Another key axis of variation is germination synchrony versus staggered germination (Maleki et al. 2023). Some species germinate en masse at the first opportunity, potentially overwhelming seed predators and maximizing exploitation of a favorable establishment window (Bogdziewicz et al. 2024). In contrast, many desert annuals and species in highly stochastic environments exhibit fractional or staggered germination as a bet‐hedging strategy, where only a fraction of seeds germinate at each opportunity while others remain dormant across subsequent years (Gremer and Venable 2014; Gremer et al. 2016; Pausas et al. 2022; Abley et al. 2024). This spreads cohorts across multiple years, ensuring that not all offspring are lost if a presumed favorable year turns unfavorable. Seed heteromorphism—where a single plant produces two or more distinct seed types with different germination properties—represents a striking extension of this strategy. In Aethionema arabicum, for example, plants produce both dehiscent fruits releasing quickly germinating seeds locally and indehiscent winged fruits that disperse further and remain dormant longer, effectively spreading regeneration in both space and time (Lenser et al. 2016; Baskin and Baskin 2014; Figure 4).

Germination speed also reflects a trade‐off with seed reserves that connects this stage directly to the seed production and seedling emergence stages of the regeneration continuum (Maleki et al. 2025; Figure 4). Small‐seeded species tend to germinate faster than large‐seeded ones, as their embryos hydrate and resume growth more readily, but they produce tiny seedlings with limited reserves that are vulnerable if conditions quickly turn unfavorable (Kadereit et al. 2017; Duncan et al. 2019; Maleki et al. 2025). Conversely, large seeds often germinate more slowly but produce robust seedlings with substantial energy reserves capable of sustaining growth through deep shade, burial, or early drought (Parsons et al. 2014; Maleki et al. 2025). As one study noted, small seeds generally germinate faster than heavy seeds, but the chance of survival and establishment at the seedling stage is greater for heavier seeds (Maleki et al. 2025). This encapsulates the core germination strategy trade‐off—quick emergence versus resilient establishment—and illustrates how germination traits are not independent of other regeneration stages but are functionally constrained by seed size decisions made during seed production and in turn determine seedling performance during emergence and early establishment.

## 4 Seedling Emergence and Soil Seed Banking

**FIGURE 5:**
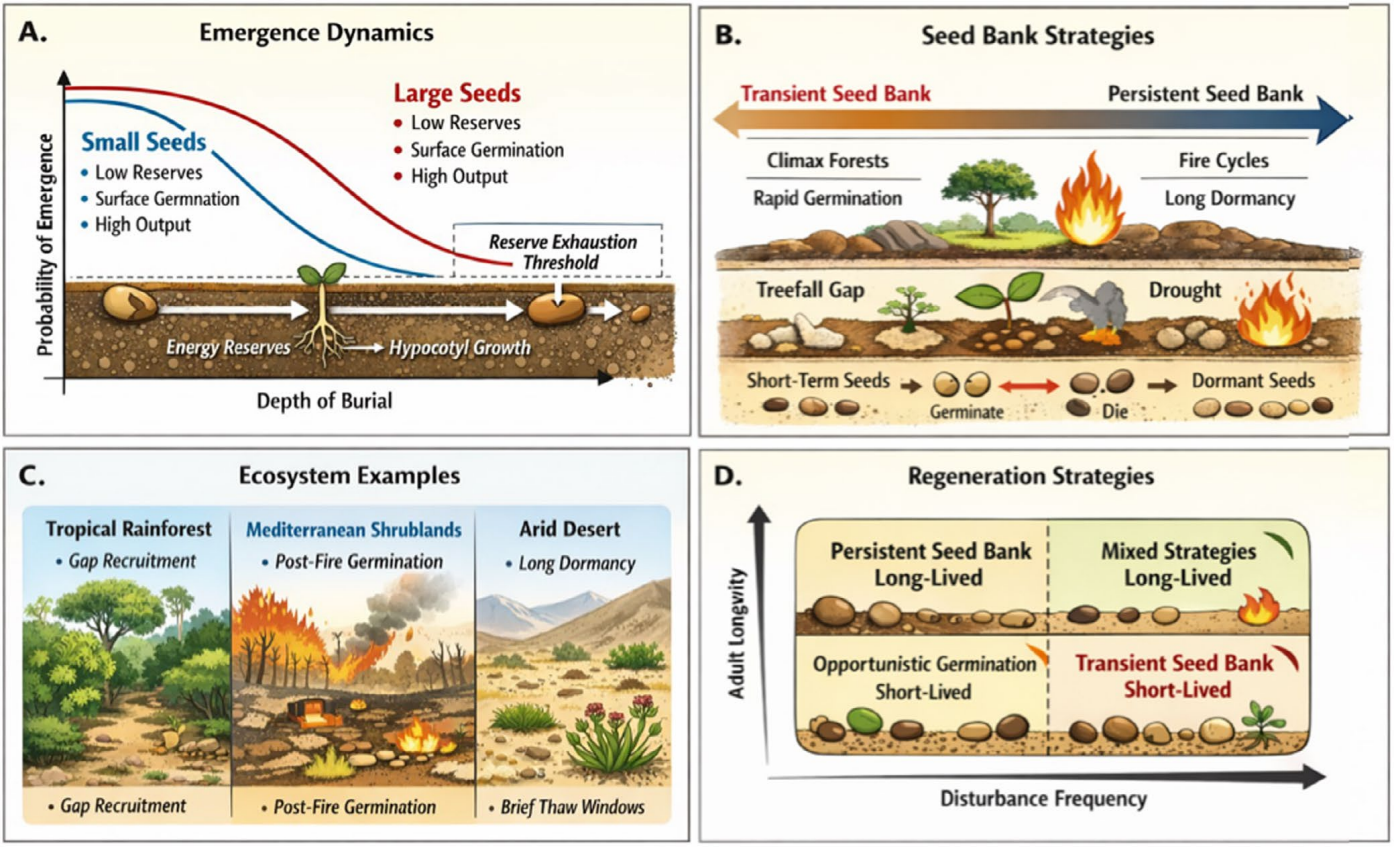
Regeneration strategies across the seed–soil continuum. (A) Emergence dynamics illustrate how seed size and burial depth influence probability of emergence through reserve availability and hypocotyl growth. (B) Seed bank strategies range from transient (rapid germination, short soil persistence) to persistent (long dormancy, multi‐year soil survival), shaped by disturbance regimes. (C) Ecosystem examples highlight contrasting regeneration modes in tropical forests, Mediterranean shrublands, and arid deserts. (D) A conceptual framework linking adult longevity and disturbance frequency shows how persistent, transient, opportunistic, and mixed strategies represent integrated regeneration syndromes across environments.

Once a seed has germinated, the next critical step in the regeneration continuum is seedling emergence, when the seedling breaks through the soil surface and becomes a photosynthetic organism (Winkler et al. 2024; Zhao et al. 2023; Figure 5). This transition links pre‐germination processes to establishment and is one of the most vulnerable phases of the plant life cycle (Vázquez‐Ramírez and Venn 2021; Kožić et al. 2024). Emergence success depends on seed traits, especially seed size and reserve allocation, as well as soil conditions, burial depth, and ecological interactions such as competition and herbivory (Figure 5). As a result, regeneration strategies at this stage are shaped by whether seedlings can reach the surface and begin autotrophic growth before reserves are exhausted and then survive the hazards of early establishment (Jørgensen et al. 2019).

Seed size and reserve allocation have a profound influence on emergence (Figure 5). Large seeds contain more stored resources, which can support elongation of the hypocotyl and early root growth, allowing seedlings to emerge from greater depths and access deeper soil moisture (Moles and Westoby 2006; Byrne et al. 2014; Ramos et al. 2017). Small seeds, by contrast, must remain close to the soil surface because even shallow burial may prevent successful emergence (Gutterman 2012). The classic trade‐off is that large seeds have a higher probability of successful emergence and seedling survival, especially in stressful environments, while small seeds, though less assured individually, can be produced in great numbers (Maleki et al. 2025). Heavier seeds also tend to perform better in shaded or competitive habitats where early growth is slow (Dalling 2024). In tropical forests, for example, understory trees often produce large, energy‐rich seeds that support seedlings in low light until disturbance creates a canopy gap (Umaña et al. 2020; Wendt et al. 2022), whereas pioneer species typically produce smaller, light‐demanding seeds and rely on high seed output to ensure that at least some reach favorable microsites (Dalling and John 2008; Umaña et al. 2020). In this way, seed production traits established earlier in the regeneration continuum strongly constrain emergence success (Dalling and John 2008; Umaña et al. 2020).

Seedling emergence is also strongly influenced by the physical and biotic environment encountered above and below ground. Soil temperature and moisture conditions at the time of germination determine whether the emerging seedling encounters a favorable or hostile environment at the soil surface (Vázquez‐Ramírez and Venn 2021). Soil surface crusting, compaction, and litter depth can all impose additional physical barriers to emergence, particularly for small‐seeded species with limited reserves (Gutterman 2012; Jørgensen et al. 2019). The mode of seedling emergence also varies among species and has important consequences for early survival. In epigeal germination, cotyledons are pushed above the soil surface and become photosynthetic, exposing them to herbivory and desiccation risk but also allowing rapid autotrophic growth. In hypogeal germination, cotyledons remain below ground as a nutrient reserve, protecting stored resources from aboveground hazards while the shoot grows upward (Gutterman 2012). This distinction is ecologically meaningful—hypogeal seedlings may be better buffered against early aboveground disturbances such as frost, fire, or herbivory, while epigeal seedlings may establish photosynthetic capacity more rapidly in high‐light environments. Once at the surface, seedlings enter a critical window between emergence and the point at which photosynthesis can sustain further growth—a period during which density‐dependent competition for light, water, and nutrients, as well as attack by herbivores and pathogens, can cause high mortality (Kožić et al. 2024). In species‐rich communities, seedlings that emerge close to the parent plant are often preferentially attacked by host‐specific enemies, further favoring those that disperse and emerge farther away—a dynamic that connects emergence directly to the dispersal strategies discussed later in this review (Comita et al. 2014; Jia et al. 2020). Together, seed size, emergence mode, and the physical and biotic environment at the soil surface define the emergence filter through which all germinated seeds must pass before contributing to population growth. In this way, seed production traits established earlier in the regeneration continuum strongly constrain emergence success, and emergence outcomes in turn determine which seedlings enter the establishment phase.

A soil seed bank, by contrast, represents the temporal alternative to immediate emergence: it is the collection of viable, ungerminated seeds present in the soil at a given time (Baskin and Baskin 2014). Within the regeneration continuum, soil seed banks store regenerative potential when conditions are unsuitable for immediate recruitment (Figure 5). Seeds enter the soil seed bank through dispersal and either germinate after a short delay (transient seed bank) or remain dormant in soil for years (persistent seed bank). The formation of a persistent seed bank is closely tied to dormancy strategies discussed earlier; seeds must be equipped (physically or physiologically) to resist decay, predation, or germination for extended periods. Persistent seed banks are particularly important in environments with episodic opportunities or frequent disturbances, and for species whose adult lifespans might be short relative to disturbance return intervals. In fire‐prone systems, some species rely on soil‐stored seeds that germinate after heat or smoke breaks dormancy, while in arid systems annuals can spread germination over multiple years and buffer populations against drought. Similar dynamics occur across biomes. In tropical rainforests, many pioneer species rely partly on persistent soil seed banks that allow recruitment when treefall gaps appear (Dalling and Brown 2009; Zalamea et al. 2025). Some tropical species show remarkable seed longevity, whereas many shade‐tolerant species lack persistent seed banks and instead rely on continuous seed rain and seedling banks in the understory (Dalling and Brown 2009; Zalamea et al. 2025; Benitez‐Malvido 2023). In grasslands and prairies, soil seed banks support recovery after fire or grazing, and many weedy forbs and grasses use them to colonize disturbed ground rapidly (Ooi et al. 2012; Eskelinen et al. 2021; Dölle and Schmidt 2009). In alpine and tundra systems, persistent seed banks can also capitalize on occasional favorable years by allowing seeds from multiple seasons to germinate when climatic windows open (Schwienbacher et al. 2010; Bernareggi et al. 2015).

Persistent seed banking, however, is not universally advantageous (Figure 5). While it offers long‐term security, keeping seeds dormant for too long can reduce a population's growth rate since those seeds are not contributing to reproduction until they germinate. If adult mortality is low and opportunities for seedling establishment are frequent, strong dormancy could even be counterproductive (Rees 1994; Postma and Ågren 2022). In many climax forest species, adult trees have long lifespans and routinely experience small canopy gaps that provide frequent microsites for seedling establishment. In such environments, most seeds germinate shortly after dispersal or lose viability within a year, resulting in predominantly transient seed banks (species whose seeds persist < 1 year; Walck et al. 2005). Conversely, in frequently disturbed or highly unpredictable habitats, such as deserts or regularly tilled fields, plants often maintain persistent seed banks, seeds that remain viable through multiple seasons, as a form of bet‐hedging against variable recruitment opportunities (Poschlod et al. 2013; Gremer and Venable 2014). Persistent seed banks buffer populations against the high risk of reproductive failure by spreading germination over many years (Baskin and Baskin 2014). Thus, the presence and duration of a soil seed bank is itself an evolved strategy aligning with a species' ecology (Etheridge and Madeira 2026). After a seed in the soil germinates, successful emergence and establishment are the final hurdles. Many seedlings face intense density‐dependent competition or natural enemy pressure (herbivores, pathogens) soon after emergence, which ties into concepts like the Janzen–Connell hypothesis in species‐rich communities where seedlings near the parent are preferentially attacked, favoring those that emerge farther away, see dispersal section (Comita et al. 2014; Jia et al. 2020).

## 5 Seed Production

**FIGURE 6:**
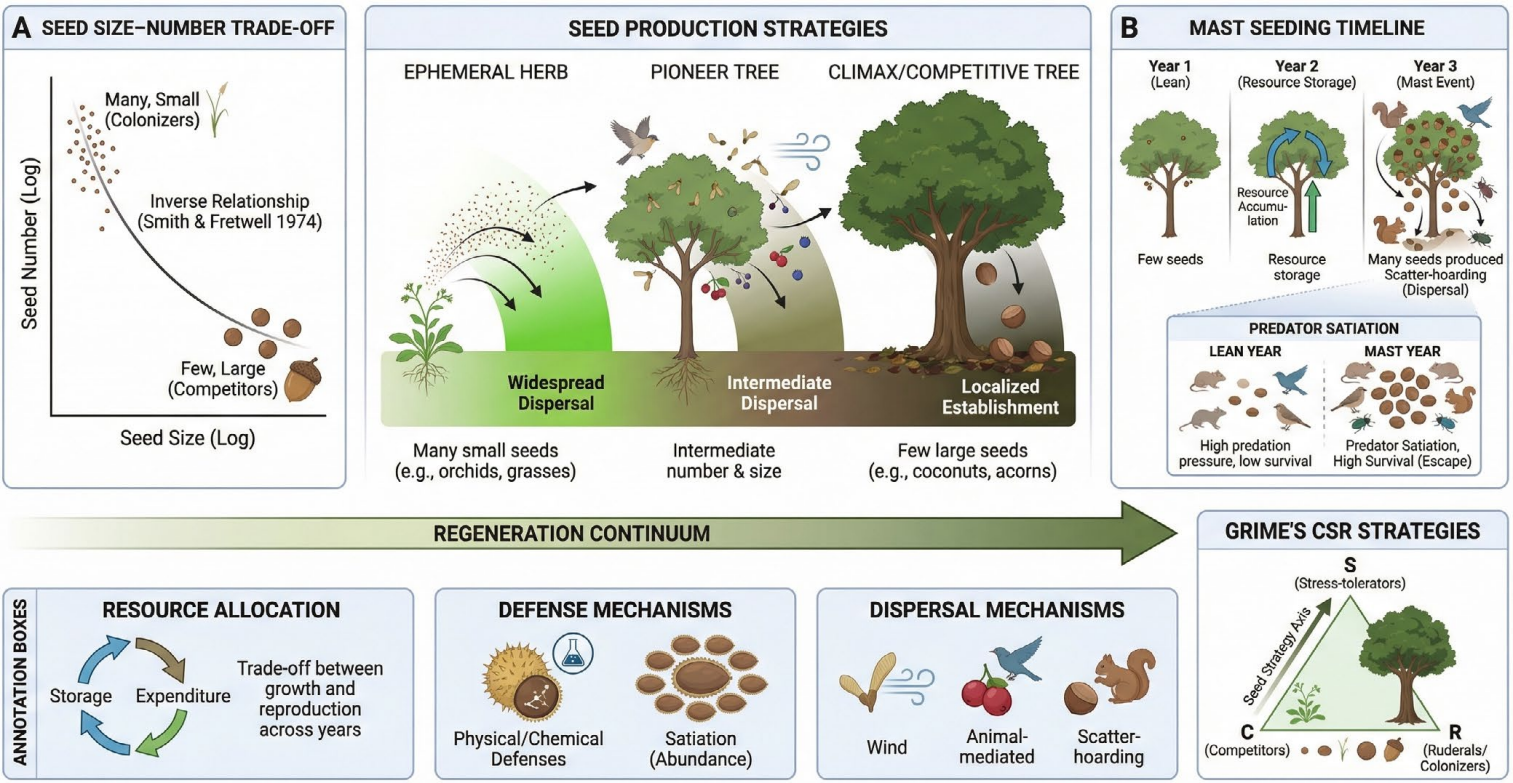
Seed production strategies and their placement along the regeneration continuum. Conceptual synthesis of the seed size–number trade‐off, contrasting many small seeds associated with colonizing strategies and few large seeds linked to competitive establishment. The figure integrates seed production syndromes (ephemeral herbs, pioneer trees, climax trees), mast seeding dynamics and predator satiation, and associated dispersal and defense mechanisms. Together, these strategies align with Grime's CSR framework and illustrate how resource allocation, dispersal mode, and reproductive timing shape regeneration outcomes across ecological contexts. This figure was created using the free version of BioRender and Figurelabs.

Seed production strategies—the patterns in which plants produce seeds in terms of quantity, size, frequency, and timing—represent the source of all propagules entering the regeneration continuum (Figure 6). Three key trade‐offs govern seed production strategies. The classic quantity–quality trade‐off (Smith and Fretwell 1974) describes how finite parental resources must be divided among offspring: producing many small seeds increases dispersal and colonization probability but reduces per‐seed provisioning, whereas producing fewer large seeds enhances individual seedling performance at the cost of reduced fecundity (Figure 6). While widely cited, empirical support for this trade‐off is surprisingly uneven, with the predicted negative relationship between seed size and number often weak or highly variable across species and ecosystems, suggesting that phylogenetic history, resource availability, and pollination system may constrain or override it in many lineages (Henery and Westoby 2001; Moles et al. 2007). The current versus future reproduction trade‐off (Williams 1966) reflects allocation decisions across time, where heavy reproductive investment in one season can reduce future survival or fecundity, though empirical demonstrations of true reproductive costs in long‐lived plants remain inconsistent, partly because resource limitation is difficult to separate from confounding factors such as weather variation or herbivory (Obeso 2002). Finally, the synchronous versus diffuse reproductive timing trade‐off (Pearse et al. 2016; Koenig 2021) concerns whether reproduction is concentrated into episodic, highly synchronized events (e.g., mast seeding) that may satiate predators and enhance pollination efficiency or spread more evenly across years to buffer against environmental unpredictability (Figure 6). However, the proximate cues and ultimate drivers of reproductive synchrony remain actively debated, and no single hypothesis has been shown to explain masting universally across species and ecosystems (Pearse et al. 2016). Together, these trade‐offs structure how species balance offspring number, offspring quality, and temporal risk in variable environments. These trade‐offs result in a wide array of reproductive syndromes across species, from ephemeral herbs that churn out thousands of tiny seeds in a single season, to large trees that invest in a few sizable seeds infrequently (Grime 1977; Moles et al. 2007; Maleki et al. 2025).

Beyond seed size and number, seed quality represents an equally important but often overlooked dimension of seed production strategy. Seed quality encompasses the biochemical composition of reserves—including the ratio of lipids to starch, protein content, and micronutrient allocation—which collectively influence germination rate, seedling vigor, and early establishment success (Fenner and Thompson 2005). Seeds with high lipid reserves generally provide more energy per unit mass than starch‐based seeds and may confer advantages in early root and shoot growth, particularly in nutrient‐poor or shaded environments (Fenner and Thompson 2005). Closely linked to seed quality are maternal effects—the influence of the mother plant's environment, physiological condition, and age on the traits of her seeds. Maternal effects can alter seed size, dormancy depth, germination timing, and seedling performance independently of the seed's own genotype (Fenner and Thompson 2005). For example, seeds produced under water stress or nutrient limitation may be smaller, have shallower dormancy, or germinate less synchronously than seeds produced under favorable conditions. Similarly, seed viability—the proportion of seeds within a population that are capable of germinating—varies considerably with pollination success, resource availability, pathogen load, and maternal plant age, and is rarely at or near 100% even in apparently healthy populations (Fenner and Thompson 2005). These dimensions of seed quality, maternal effects, and viability mean that the propagule pool entering the regeneration continuum is highly heterogeneous in quality, not just quantity, with important but often underappreciated consequences for subsequent dormancy, germination, and establishment outcomes.

The seed size–number trade‐off has direct consequences for subsequent regeneration stages (Figure 6). Small seeds, produced in large numbers, enhance the probability that some will reach favorable microsites, disperse farther, and colonize open or disturbed areas (Beckman and Sullivan 2023), but each seedling has limited reserves and a lower chance of individual survival (Soltani et al. 2025). Large seeds are more expensive to produce but provide each seedling with a competitive advantage during emergence and early establishment, particularly in shaded or stressful environments (Vandelook et al. 2018). However, the advantage of large seeds diminishes in disturbed or resource‐rich environments, and seedling survival often depends more strongly on microsite conditions than on seed size per se (Leishman et al. 2000). The competitive advantage hypothesis for seed size—that species in resource‐poor or highly competitive environments tend to produce larger seeds (Leishman et al. 2000; Moles and Westoby 2004)—has been documented across biomes but has also been challenged, as the relationship weakens when phylogenetic relatedness is controlled for, raising the possibility that observed patterns partly reflect evolutionary history rather than direct adaptation (Moles and Westoby 2004). This spectrum is evident among tropical trees, where fast‐growing pioneer gap colonizers produce small, wind‐ or bird‐dispersed seeds, while climax species produce larger seeds capable of establishing in low‐light understory conditions. Seed production strategy thus aligns with the regeneration niche, with colonizers and competitors representing opposite ends of the size–number continuum, echoing plant strategy theory (Laughlin 2023; Figure 6).

Mast seeding represents the most striking example of temporal variation in seed production (Pearse et al. 2016). Masting, where perennials produce very large numbers of seeds in certain years but little or none in others, often synchronously across large areas (Bogdziewicz et al. 2018), is common in many forest ecosystems including oaks, hardwoods, conifers, and dipterocarp rainforest trees (Koenig 2021; Bogdziewicz 2022; Fleurot et al. 2023). Despite decades of research, the proximate triggers of masting remain controversial, with competing resource matching, weather cue, and carbon‐nutrient balance hypotheses that are not mutually exclusive and whose relative importance likely varies among species and ecosystems (Pearse et al. 2016; Bogdziewicz et al. 2018). Among the proposed evolutionary benefits, predator satiation is most frequently cited: boom‐and‐bust seed supply dynamics reduce predator populations in lean years, causing per‐seed predation to drop dramatically in mast years even as absolute predator numbers rise (Bogdziewicz et al. 2018; Koenig 2021). However, this hypothesis has been questioned on theoretical and empirical grounds, as predator populations can in some systems track mast events closely enough to erode the satiation benefit over time (Kelly and Sork 2002). Masting may also improve pollination efficiency in wind‐pollinated species through pollen economies of scale, and enhance animal‐mediated dispersal by triggering scatter‐hoarding behavior in rodents and birds that bury far more seeds than they can consume (Bogdziewicz et al. 2018). While the pollination efficiency benefit is intuitively appealing, empirical evidence linking masting to improved fertilization success is less consistent than for predator satiation (Pearse et al. 2016). Resource allocation underlies these patterns: many perennials accumulate reserves in lean years before investing heavily in mast years, consistent with the resource storage hypothesis, though this has been challenged by evidence that temperature differentials between years may be more reliable masting predictors than internal resource dynamics (Bogdziewicz et al. 2020). The relative contributions of internal resource regulation and external environmental cues to masting therefore remain unresolved and likely differ among species (Pearse et al. 2016; Bogdziewicz et al. 2020).

Finally, seed predation pressure shapes production strategies through investment in chemical or physical seed defenses such as tannins or thick coats. However, chemical defenses that deter predators may simultaneously reduce seed attractiveness to mutualistic dispersers, creating an unresolved conflict between defense and dispersal functions that has rarely been formally modeled across species (Janzen 1971). The conditions under which seed defense is favored over alternative strategies such as predator satiation or dispersal‐based escape remain an open question (Janzen 1971). Seed production strategies thus do not operate in isolation: the quantity, size, timing, and defense of seeds produced at this stage directly determine the propagule pool available for dispersal, soil seed banking, germination, and seedling emergence, linking this stage to all other components of the regeneration continuum.

## 6 Dispersibility

**FIGURE 7:**
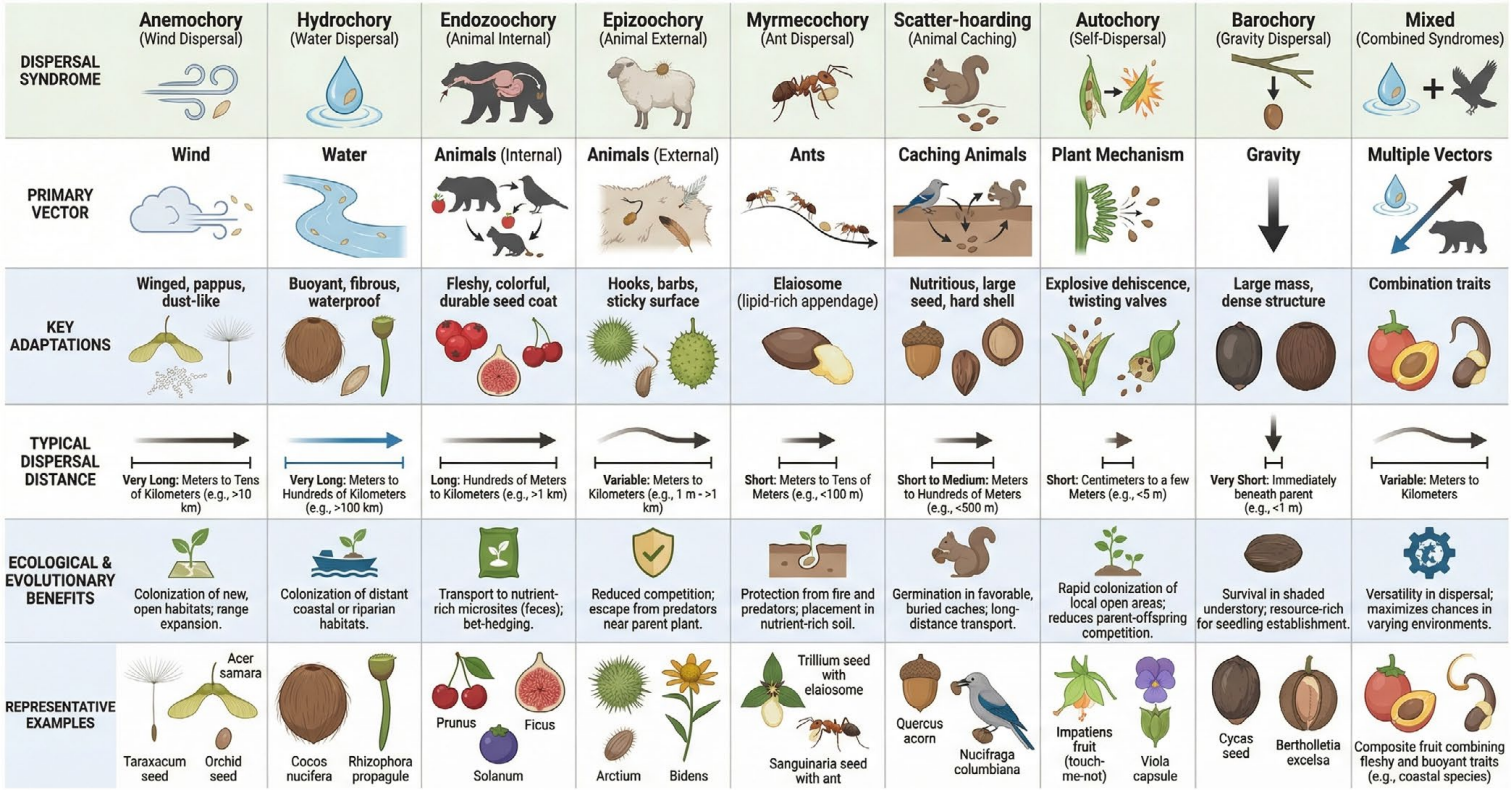
Major seed dispersal syndromes and their ecological functions. Overview of primary dispersal modes: anemochory, hydrochory, endozoochory, epizoochory, myrmecochory, scatter‐hoarding, autochory, barochory, and mixed strategies, highlighting vectors, key morphological adaptations, typical dispersal distances, ecological benefits, and representative examples. This figure was created using the free version of BioRender and Figurelabs.

Dispersal represents a critical stage of the regeneration continuum, determining not just how far seeds travel but where in space regeneration can potentially occur (Figure 7). Dispersibility—the capacity of seeds to be carried by various vectors including wind, water, and animals—is fundamentally a measure of a species' spatial regeneration potential: the range of microsites, habitats, and geographic areas that propagules can reach and within which germination, emergence, and establishment may follow (Aslan et al. 2019; Beckman and Sullivan 2023). By governing the spatial placement of propagules, dispersal connects seed production to all subsequent regeneration stages—a seed can only germinate, emerge, and establish where it lands. Effective dispersal therefore expands the realized regeneration niche in space, reduces parent‐offspring competition, enables colonization of new or disturbed habitats, and allows escape from the concentration of host‐specific predators and pathogens near the parent, as formalized in the Janzen‐Connell hypothesis (Aslan et al. 2019; Beckman and Sullivan 2023; Davies et al. 2025). However, the actual fitness benefits of dispersal are difficult to quantify empirically, and there is ongoing debate about whether dispersal limitation or habitat limitation is the primary constraint on plant distribution and recruitment in most ecosystems (Pulliam 2000; Nathan and Muller‐Landau 2000). Furthermore, long‐distance dispersal events, while ecologically important for range expansion and colonization, are rare and stochastic, making them difficult to incorporate reliably into predictive models of plant spread or community assembly (Nathan and Muller‐Landau 2000). Many empirical studies have documented distance‐ and density‐dependent seedling survival consistent with the Janzen‐Connell framework, suggesting that dispersal away from the parent can enhance recruitment success and maintain species diversity by preventing competitive dominance (Comita et al. 2014; Jia et al. 2020; Detto and Pacala 2024; Magee et al. 2025).

Plants have evolved an astonishing variety of dispersal mechanisms, each suited to particular environments and life histories (Figure 7). Broadly, the major dispersal syndromes include anemochory (wind dispersal), hydrochory (water dispersal), zoochory (animal dispersal)—which can be subdivided into endozoochory (inside an animal, typically via ingestion and defecation) and epizoochory (external transport on animal bodies), as well as autochory (self‐dispersal) mechanisms like ballistic ejection or gravity drop (Figure 7). Many species combine multiple modes (e.g., a fruit that falls by gravity and then is moved by animals, or a seed that can float but also be carried by animals).

Wind dispersal is one of the most common strategies, especially in open habitats and among pioneer species (Beckman and Sullivan 2023; Brightly et al. 2024). Wind‐dispersed seeds have morphological adaptations including wings (e.g., samaras of maples and dipterocarps, winged conifer seeds), plumes or silky hairs (e.g., pappus of dandelion, cottony hairs of willow and poplar), or very low mass (dust seeds of orchids), all of which increase aerodynamic lift or drag (Brightly et al. 2024; Figure 7). In deserts and grasslands, some plants produce tumbleweeds that detach and roll in the wind, shedding seeds over large distances (Stallings et al. 1995; Borger et al. 2007). While wind dispersal can facilitate long‐distance colonization—volcanic islands, for example, are often initially colonized by wind‐borne propagules (Nathan et al. 2008; Nogales et al. 2012)—mechanistic models and empirical studies suggest that the vast majority of wind‐dispersed seeds land close to the parent plant, with only a small tail of the dispersal kernel reaching truly long distances (Nathan et al. 2002). This discrepancy between potential and realized dispersal distances means wind dispersal may be less effective at facilitating range expansion than is often assumed (Nathan et al. 2002). Notable cases include *Pinus contorta* , whose serotinous cones release winged seeds after fire to help re‐vegetate burns, and Asian dipterocarps, whose large winged seeds can glide 50–100 m in strong winds to escape the parent's shade and pest zone (Smith et al. 2015). The high propagule output required to offset dispersal inefficiency also reinforces the seed size‐number trade‐off discussed in the seed production section, and whether wind‐dispersed species achieve higher colonization success than animal‐dispersed species with fewer but more targeted seeds remains an unresolved question (Nathan et al. 2002).

Water dispersal (hydrochory) is crucial for plants in aquatic or riparian environments and for some coastal species (Nilsson et al. 2010). Aquatic plants often have seeds or fruits that float, using water currents to move (Fraaije et al. 2017). For instance, the seeds of many sedges and rushes have air‐filled tissues. Mangroves have viviparous propagules (e.g., Rhizophora seedlings are buoyant pencils that drift on tides until lodging in mud to root) (Van der Stocken et al. 2019). Perhaps the most famous hydrochorous seed is the coconut ( *Cocos nucifera* ): coconuts are adapted to float in seawater, protected by a fibrous husk, and can travel hundreds of kilometers across oceans to germinate on distant shores, a powerful strategy for island colonization (Beveridge et al. 2022). While the coconut is frequently cited as an example of long‐distance hydrochory, it is worth noting that the relative contributions of natural ocean dispersal versus human‐mediated transport to the current pantropical distribution of coconuts remain debated (Baudouin and Lebrun 2009). This example highlights a broader challenge in dispersal ecology: distinguishing between natural dispersal capacity and historically human‐influenced distributions, particularly for species with long associations with human civilizations (Baudouin and Lebrun 2009). Rivers can disperse seeds within continents; many tropical riverine species have floating fruits (like the cannonball tree Couroupita or certain palms) (Nilsson et al. 2010). In seasonal floodplains, water dispersal ensures seeds reach safe sites once floods recede. One interesting adaptation is in Neotropical swamp forests where some seeds will only germinate after being submerged for weeks (a cue that water has transported them to a suitable site that will soon dry) (Parolin et al. 2013).

Animal dispersal generates the most diverse and spatially complex regeneration templates, ranging from short‐distance targeted deposition to rare long‐distance events (Beckman and Sullivan 2023; Figure 7). Critically, animal‐mediated dispersal is not only about distance but about the quality of spatial placement—where seeds are deposited relative to suitable germination microsites determines whether dispersal actually translates into regeneration potential (Schupp et al. 2010). Dispersal effectiveness depends not only on the number of seeds moved but also on deposition location and establishment probability at those sites, components that are rarely measured simultaneously, meaning seed removal rates used as proxies can be highly misleading (Schupp et al. 2010). In endozoochory, gut passage by birds and mammals transports seeds to new locations, potentially across gaps or rivers, though most seeds are deposited relatively close to fruiting trees and the contribution of truly long‐distance events to spatial regeneration potential remains poorly quantified (Jordano 2000; Viana et al. 2016; Corlett 2017; Bracho‐Estévanez et al. 2025). Some plants time fruiting to animal migration seasons, effectively using migratory species as vectors for regional‐scale spatial regeneration (Jordano 2000; Herrera and Garcia 2010). Scatter‐hoarding by squirrels, jays, and nutcrackers generates spatially explicit regeneration opportunities at cache sites often located in habitats favorable for seedling establishment, as in the well‐documented mutualism between Clark's nutcracker and whitebark pine where seeds are transported up to 10–15 km across mountain valleys to cached sites (Vander Wall 2001; Gómez 2003; Lorenz et al. 2011; Pesendorfer et al. 2016). Epizoochory via hooks, barbs, or sticky secretions generates local to medium‐range spatial regeneration potential along animal movement corridors (Gutterman 2012; Heinken et al. 2006; Sorensen 1986; Will and Tackenberg 2008; Albert et al. 2015; Figure 7). Myrmecochory generates highly targeted short‐distance spatial regeneration in nutrient‐rich ant nest microsites, though net fitness advantages of this precise placement remain contested across systems (Lengyel et al. 2010; Devenish and Gómez 2021; Giladi 2006; Figure 7). Ballistic dispersal ensures seeds escape the immediate vicinity of the parent, generating a modest but reliable local expansion of spatial regeneration potential, particularly effective in dense vegetation where wind is limited.

Fruits and frugivores have often evolved in tandem, with fruiting times, colors, and nutrients matching animal preferences and animals evolving tolerance or attraction to specific fruit chemistries. A well‐known example is the dispersal of chili pepper seeds (Capsicum) exclusively by birds: birds are immune to capsaicin and disperse seeds intact, whereas mammalian seed predators are deterred by the heat—an elegant selective filter that illustrates how coevolution can shape both plant chemistry and disperser specificity (Tewksbury and Nabhan 2001; Tewksbury et al. 2008). The directed dispersal hypothesis further suggests that some dispersers deposit seeds preferentially in favorable microsites—ants to nutrient‐rich nests, jays to open sunny patches—improving seedling success beyond what random deposition would achieve. However, many plant‐disperser relationships are facultative rather than obligate and are highly sensitive to disruption. The ongoing loss of large frugivores—including megafauna, large primates, and large birds—is already causing dispersal failure for large‐seeded plants that evolved with now‐extinct or locally extirpated partners (Galetti et al. 2013), with cascading consequences for forest regeneration and community composition that are only beginning to be quantified (Galetti et al. 2013; Beckman, Aslan, and Rogers 2020). Whether plant populations can adapt to alternative dispersers when primary mutualists are lost remains largely unknown and represents a critical frontier for dispersal ecology and conservation biology (Beckman, Aslan, and Rogers 2020). Dispersal therefore closes the regeneration continuum not merely by determining how far seeds travel, but by governing where propagules are deposited, whether ecological partnerships remain intact, and ultimately whether the next generation of plants can successfully enter the germination, emergence, and establishment stages that complete the cycle.

## 7 Regeneration Syndromes Across Environmental Gradients: A Synthesis

**FIGURE 8:**
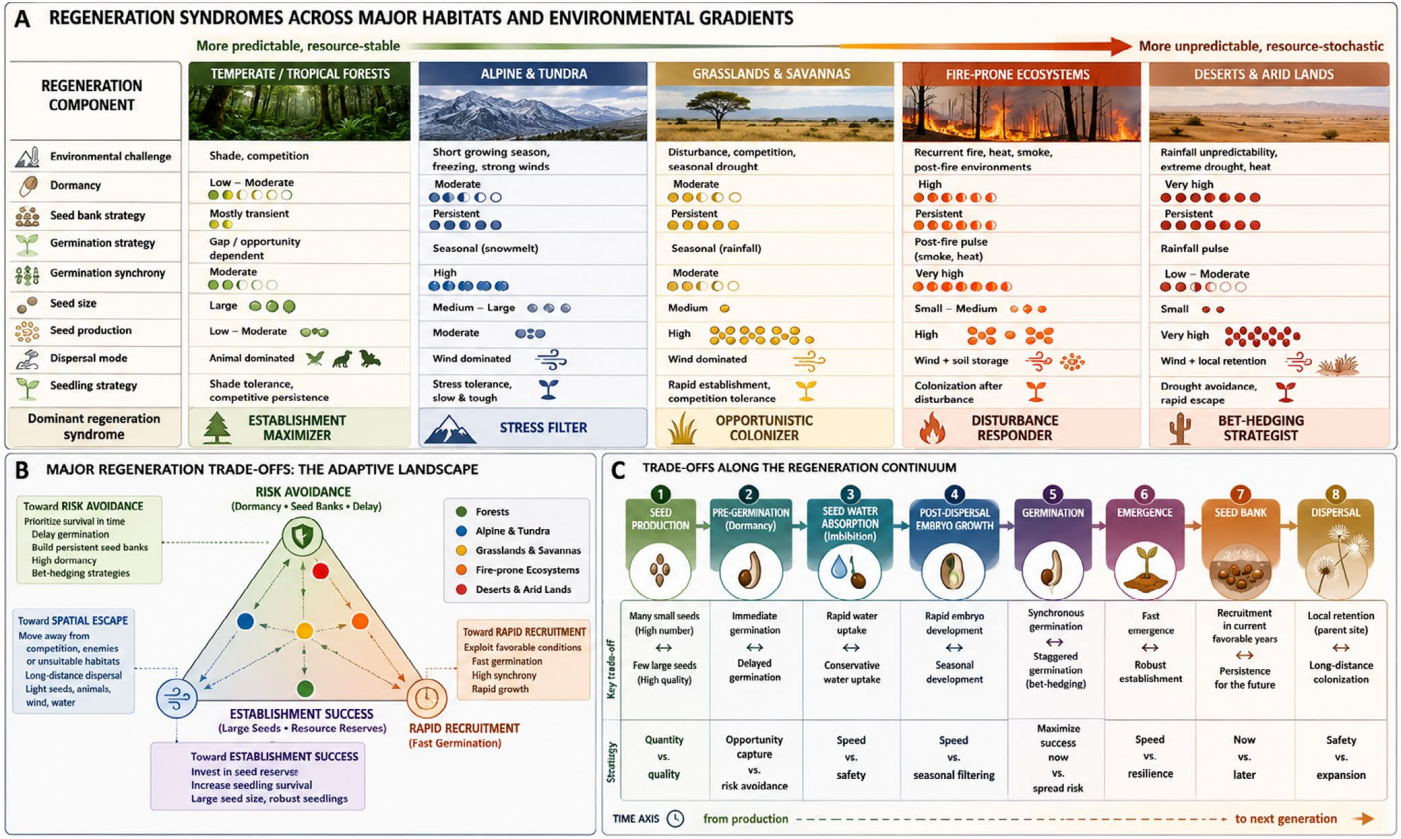
Regeneration syndromes and trade‐offs across environmental gradients. Regeneration strategies in seed plants can be viewed as coordinated trait syndromes that recur across habitats and environmental regimes. (A) Major regeneration syndromes associated with forests, alpine systems, grasslands, fire‐prone ecosystems, and deserts. (B) Conceptual adaptive landscape illustrating how regeneration strategies balance risk avoidance, rapid recruitment, establishment success, and spatial escape. (C) Key trade‐offs operating across successive stages of the regeneration continuum. Together, these patterns illustrate how distinct combinations of dormancy, germination, emergence, seed banking, seed production, and dispersal traits represent alternative solutions to the common challenge of maximizing recruitment under environmental uncertainty.

Although regeneration traits are often studied individually, the framework developed here suggests that they are frequently assembled into coordinated regeneration syndromes that recur across environmental gradients (Figure 8). Species occupying similar ecological contexts often converge on comparable combinations of dormancy, germination, emergence, seed banking, seed production, and dispersal traits because they face similar constraints on recruitment success. These syndromes can be viewed as alternative solutions to a common challenge: balancing the trade‐off between risk avoidance and opportunity capture during regeneration. At one end of the spectrum, species from relatively stable and predictable environments, such as many temperate and tropical forests, tend to emphasize establishment success. These species commonly produce larger seeds, invest heavily in seedling performance, maintain transient seed banks, and rely on animal‐mediated dispersal to place propagules in favorable microsites. In contrast, species from highly stochastic environments, particularly deserts and arid ecosystems, often exhibit regeneration syndromes characterized by strong dormancy, persistent seed banks, rapid germination following rainfall, and high seed production. Together, these traits spread recruitment risk across time and reduce the likelihood of complete reproductive failure during unfavorable years. Intermediate syndromes occur in other environments. Alpine species frequently combine seasonal dormancy and stress‐tolerant seedlings, reflecting short growing seasons and harsh climatic conditions. Grassland species often exhibit opportunistic colonization strategies characterized by moderate dormancy, persistent seed banks, high fecundity, and wind dispersal. In fire‐prone ecosystems, regeneration syndromes are strongly linked to disturbance, with serotiny, fire‐cued germination, persistent seed storage, and rapid post‐fire recruitment commonly occurring together. These patterns suggest that regeneration strategies can be understood within a multidimensional adaptive landscape shaped by three broad selective pressures: risk avoidance, rapid recruitment, and establishment success (Figure 8). Different regeneration stages contribute to these axes through a series of interconnected trade‐offs, including immediate germination versus delayed germination, rapid versus conservative water uptake, synchronous germination versus bet‐hedging, recruitment today versus persistence through seed banking, and local retention versus long‐distance dispersal. Because these trade‐offs operate sequentially throughout the regeneration continuum, evolutionary changes at one stage often constrain or reinforce strategies at subsequent stages. Viewing regeneration through the lens of coordinated syndromes provides a useful bridge between trait‐based ecology, life‐history theory, and regeneration biology. It helps explain why similar trait combinations repeatedly evolve in unrelated lineages occupying comparable environments and offers a predictive framework for understanding how regeneration strategies may shift under changing climates and disturbance regimes.

## 8 Concluding Remarks

Regeneration in seed plants is best understood not as a set of isolated traits, but as a coordinated continuum that links developmental timing, establishment filters, and spatial placement. Across biomes, predictable suites of traits recur because failure at any stage—hydration, dormancy release, embryo completion, germination cueing, emergence, seed banking, fecundity, or dispersal—terminates recruitment. Framing regeneration as an integrated strategy clarifies why seemingly opposite solutions (e.g., rapid germination vs. prolonged dormancy; many small seeds vs. few large seeds; local retention vs. long‐distance dispersal) can each be adaptive: they resolve the same fundamental trade‐off between risk avoidance and opportunity capture, but under different regimes of environmental variability, disturbance, competition, and enemy pressure. This continuum perspective also helps reconcile classic ecological dichotomies (colonizer–competitor, ruderal–stress tolerant) by showing how temporal buffering (dormancy, seed banks, masting) and spatial escape (dispersal modes, directed dispersal, Janzen–Connell processes) combine to generate coherent regeneration syndromes. An explicit continuum framework yields testable predictions. For example, persistent seed banks and fractional germination should co‐occur with high interannual recruitment variance and short adult longevity, whereas transient seed banks and rapid recruitment should dominate where establishment opportunities are frequent and adult survival is high. Likewise, traits that govern the earliest “commitment” steps—seed water uptake dynamics and post‐dispersal embryo growth—should strongly structure germination niches and climate sensitivity, yet remain underrepresented in comparative datasets and models. By integrating these early filters with emergence constraints, seed supply dynamics, and dispersal placement, the regeneration continuum provides a mechanistic bridge from individual traits to population persistence, community assembly, and macroevolutionary diversification. This synthesis is especially timely under global change. Shifts in precipitation regimes, altered disturbance cycles, habitat fragmentation, and changing mutualist–enemy landscapes will act most strongly on regeneration stages and may decouple historically aligned trait packages from contemporary conditions. We suggest three priorities for next steps: (i) building cross‐biome, phylogenetically explicit datasets that include imbibition and embryo‐growth traits alongside dormancy, seed banks, and dispersal; (ii) embedding regeneration continua into demographic and distribution frameworks that explicitly model stage‐specific bottlenecks and safe‐site limitation; and (iii) testing how coordinated regeneration syndromes mediate climate vulnerability and resilience. Treating regeneration as a continuum moves the field beyond trait‐by‐trait explanations and toward a unified, predictive ecology of how plants persist, coexist, and adapt in a rapidly changing world.

## Author Contributions

**Keyvan Maleki:** conceptualization (equal), visualization (equal), writing – original draft (equal), writing – review and editing (equal). **Elias Soltani:** conceptualization (equal), visualization (equal), writing – original draft (equal), writing – review and editing (equal).

## Funding

The authors have nothing to report.

## Conflicts of Interest

The authors declare no conflicts of interest.

## Data Availability

This article is a Review and does not report original experimental data or generate new datasets. All information discussed is derived from previously published literature, which has been appropriately cited within the manuscript. Therefore, no additional data are available.
